# Iterative User Interface Design for Automated Sequential Organ Failure Assessment Score Calculator in Sepsis Detection

**DOI:** 10.2196/humanfactors.7567

**Published:** 2017-05-18

**Authors:** Christopher Ansel Aakre, Jaben E Kitson, Man Li, Vitaly Herasevich

**Affiliations:** ^1^ Mayo Clinic Department of Medicine, Division of General Internal Medicine Rochester, MN United States; ^2^ Mayo Clinic Department of Information Technology Rochester, MN United States; ^3^ Mayo Clinic Multidisciplinary Epidemiology and Translation Research in Intensive Care (METRIC) Rochester, MN United States; ^4^ Mayo Clinic Department of Anesthesia and Perioperative Medicine Rochester, MN United States

**Keywords:** automation, organ dysfunction scores, software design, user-computer interface

## Abstract

**Background:**

The new sepsis definition has increased the need for frequent sequential organ failure assessment (SOFA) score recalculation and the clerical burden of information retrieval makes this score ideal for automated calculation.

**Objective:**

The aim of this study was to (1) estimate the clerical workload of manual SOFA score calculation through a time-motion analysis and (2) describe a user-centered design process for an electronic medical record (EMR) integrated, automated SOFA score calculator with subsequent usability evaluation study.

**Methods:**

First, we performed a time-motion analysis by recording time-to-task-completion for the manual calculation of 35 baseline and 35 current SOFA scores by 14 internal medicine residents over a 2-month period. Next, we used an agile development process to create a user interface for a previously developed automated SOFA score calculator. The final user interface usability was evaluated by clinician end users with the Computer Systems Usability Questionnaire.

**Results:**

The overall mean (standard deviation, SD) time-to-complete manual SOFA score calculation time was 61.6 s (33). Among the 24% (12/50) usability survey respondents, our user-centered user interface design process resulted in >75% favorability of survey items in the domains of system usability, information quality, and interface quality.

**Conclusions:**

Early stakeholder engagement in our agile design process resulted in a user interface for an automated SOFA score calculator that reduced clinician workload and met clinicians’ needs at the point of care. Emerging interoperable platforms may facilitate dissemination of similarly useful clinical score calculators and decision support algorithms as “apps.” A user-centered design process and usability evaluation should be considered during creation of these tools.

## Introduction

As electronic medical records (EMRs) have propagated through the US health care system, they have brought both great promise and great problems [1,2]. One unintended consequence of increasing EMR adoption that has been recently characterized is physician burnout associated with EMR-associated clerical tasks [3]. The high clerical burden of these tasks may be a consequence of variable attention given to usability and user-centered design by vendors [4,5]. Health information technology interfaces that are not well adapted to clinician workflow can both increase clerical workload and potentially pose safety risks to patients [6-8]. As in other industries, medicine has sought to overcome task-related inefficiencies through automation [9].

Automation of computer interaction in clinical medicine can take many forms. Automated information retrieval is commonly utilized to generate shift hand-off and inpatient rounding reports, significantly reducing time spent on information retrieval tasks [10-12]. Automating clinical guideline implementation through clinical decision support rules has also been done to reduce practice variability by promoting standards of care [13,14]. A recent change in the definition of sepsis has opened a challenge to create and implement clinical decision support that could reduce the clinician workload of information retrieval and processing specific to the sequential organ failure assessment (SOFA) score [15].

In March 2016, the operational definition of sepsis was updated to include a change in SOFA score ≥2 compared with baseline (ΔSOFA) [15]. The updated definition has been controversial [16-20]. The SOFA score, which assesses organ dysfunction in six domains, was created in 1996 to describe sepsis-related organ dysfunction [21]. Originally, the SOFA score was calculated at admission [21]. With time, usage has been extended to include serial recalculation using the most abnormal values during the preceding 24 h [22]. However, the new sepsis definition suggests that the SOFA score would need more frequent recalculation to identify sepsis in real time.

The prospective time-drain imposed by the new definition may not be trivial; previous studies have indicated a time-cost of about 5 min for information retrieval and manual score calculation per patient [23]. Consequently, methods to include automated SOFA score calculations in daily clinical reports have been created [24,25]. EMR interfaces have advanced since those studies and the time-drain of manual SOFA calculation may have changed. Additionally, these previous automated SOFA score calculators were used in printed daily reports and have not been adapted to meet clinician needs for real-time use at the bedside.

The goals of this study were to (1) quantify the current time-drain of manual SOFA score calculation and (2) describe the user-centered design process and usability evaluation of an EMR-integrated real-time automated SOFA score calculator interface.

## Methods

### Setting

This study was performed at Mayo Clinic Hospital, St Marys Campus in Rochester, Minnesota. The study protocol was reviewed and approved by the Mayo Clinic Institutional Review Board.

### Time-Motion Analysis

Internal medicine residents were observed calculating baseline and current SOFA scores during their medical intensive care unit (ICU) rotation over a 2-month period. Residents utilized Mayo Clinic’s locally developed EMR for data retrieval. The instrument (website, mobile phone app, etc) utilized to perform the calculation was at the clinician's discretion. Total calculation time and calculation instrument were captured for each observation. The total time-cost was calculated using average task completion time, assuming one SOFA score calculation or patient day, and extrapolated to the total number of patient medical ICU days at St Marys Hospital in Rochester, MN during a 1-year period.

### Interface Development and Usability Evaluation

The user interface was designed using an agile development process involving stakeholders from critical care medicine and information technology. Agile software development is a user-centered design process where programs are built incrementally in many short development cycles. These development cycles are analogous to plan-do-study-act cycles utilized in clinical quality improvement. In contrast with traditional “waterfall” linear software development, end-user testing and feedback is performed during each agile developmental cycle rather than during the last phase of the project. Agile software development utilizes close collaboration between developers and end users to guide improvements during each cycle—this feature allows early customization of the user interface (UX) to meet the clinician end user’s information needs. Close involvement of clinician end users throughout the development process has been shown to improve usability and end-user utilization of the resulting product [26,27].

**Figure 1:**
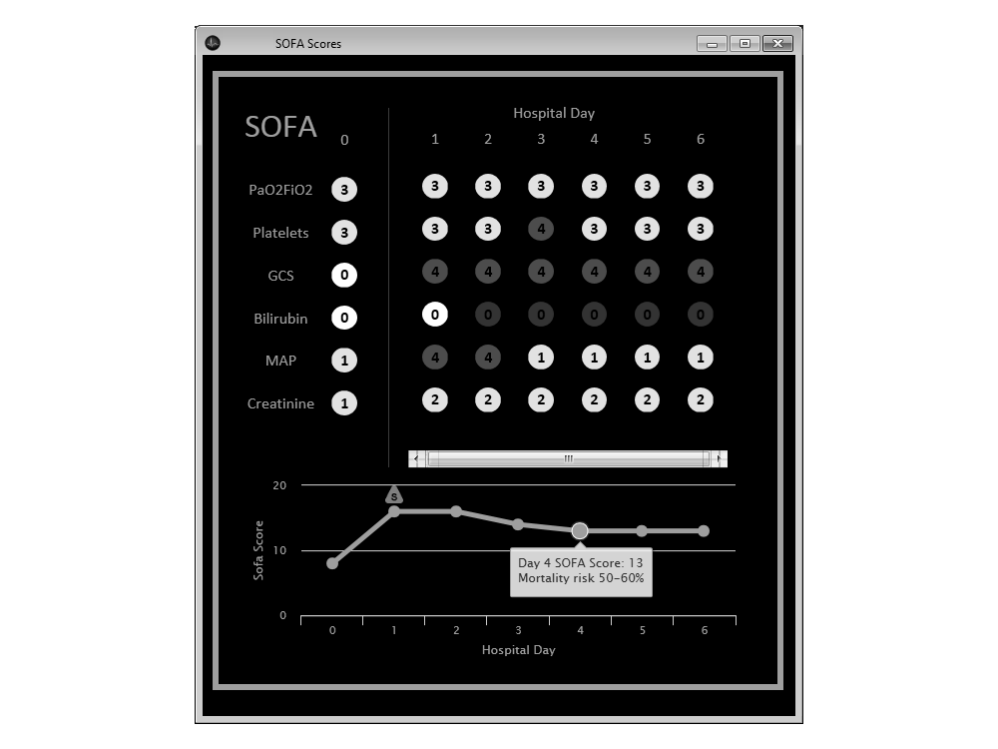
Example of the automated sequential organ failure assessment (SOFA) score calculator’s final implemented user interface.

The algorithm underlying the SOFA score calculator was previously validated for daily score calculation [25] and updated to facilitate more frequent recalculation every 15 min. With each recalculation, the 24-h calculation frame is shifted by 15 min. During the initial planning phase, clinician stakeholders were interviewed to determine essential and nice-to-have user interface features and how to display information for each SOFA subcomponent. Next, a UX mockup was constructed using Pencil (Evolus, Ho Chi Min City, Vietnam), an open-source multi-platform graphical user interface (GUI) prototyping tool, and returned to clinician stakeholders for review and comment. To complete the cycle, changes were made to the UX prototypes by developers and returned to clinicians for review and feedback. We continued iterative UX development cycles until a consensus was reached on interface design. The interface underwent a total of four iterative development cycles spanning 2 weeks before consensus was reached. The final UX was integrated into clinical workflow through our institution’s ICU patient care dashboard by adding indicator icons to our unit-level multipatient viewer. The indicator icon changes when the ΔSOFA criteria have been met but does not trigger a visual alert. A mouse-click on the indicator displays the automated SOFA score calculator interface (Figure 1).

The final UX was evaluated with the Computer Systems Usability Questionnaire administered through REDCap [28,29]. The questionnaire was sent to all potential end users not involved in UX development who were scheduled to work during the 2 months after the interface had been made available for clinical use. A 5-point Likert scale was used for each item. Responses to each item were categorized as favorable (4-5), neutral (3), or unfavorable (1-2). Each question item belonged to one of three domains—system usability, information quality, and interface quality [28]. The proportion of question items categorized as favorable, neutral, and unfavorable was calculated for the overall questionnaire and within each domain.

All statistical analysis for study was performed with R version 3.3.1 [30]. For the time motion analysis, linear regression was performed to assess the relationship between hospital day and calculation time. Descriptive statistics were used to describe the survey participants’ clinical roles and the proportion of responses within each usability domain.

## Results

### Time-Motion Analysis

Fourteen internal medicine residents were observed calculating 35 baseline and 35 current SOFA scores for patients admitted to the medical ICU under their care. The overall mean (SD) calculation time was 61.6 s (33). The time required to calculate the current SOFA score was significantly lower than the baseline score (39.9 s [8.3] vs 83.4 s [36.0]; *P*<.001). Most participants (9/14, 64%) manually entered data points into a Web-based score calculator; the remainder used a mobile phone app. There was a significant linear association between current hospital day and time to calculate baseline score (*P*<.001, R^2^=.54). If we extrapolated the time-cost to an entire year within our institution’s 24-bed medical intensive care unit, the cumulative time required for one extra manual SOFA score calculation for each patient day (6770 patient days) would be about 116 (64) hours. This amounts to almost 5 extra hours of work per ICU bed distributed among our medical intensive care clinicians.

### Interface Design and Usability Evaluation

Clinician stakeholders identified several key features during the initial stakeholder analysis. Essential needs identified by clinicians reflected their clinical information needs: (1) ability to quickly identify when the ΔSOFA≥2 (vs baseline) threshold had been passed; (2) ability to quickly view current, baseline, and previous SOFA scores from the current hospitalization, broken down by SOFA score component; (3) ability to quickly identify when data was missing for each SOFA component and if data was carried forward; (4) ability to quickly identify the source data used for each SOFA component calculation; and (5) high accuracy. Items 3-5 reflect the concerns several stakeholders expressed about the potential for automation bias with this tool [31]. One nonessential need was identified: Displaying prognostic mortality risk associated with each SOFA score. All identified information needs were incorporated into the initial UX mockup. Major UX changes during the development process included (1) formatting and coloring changes to highlight extreme or missing data for each SOFA component, (2) changes to the ΔSOFA threshold indicator, and (3) changes to limit the quantity of daily SOFA scores visible for prolonged hospitalizations.

**Figure 2:**
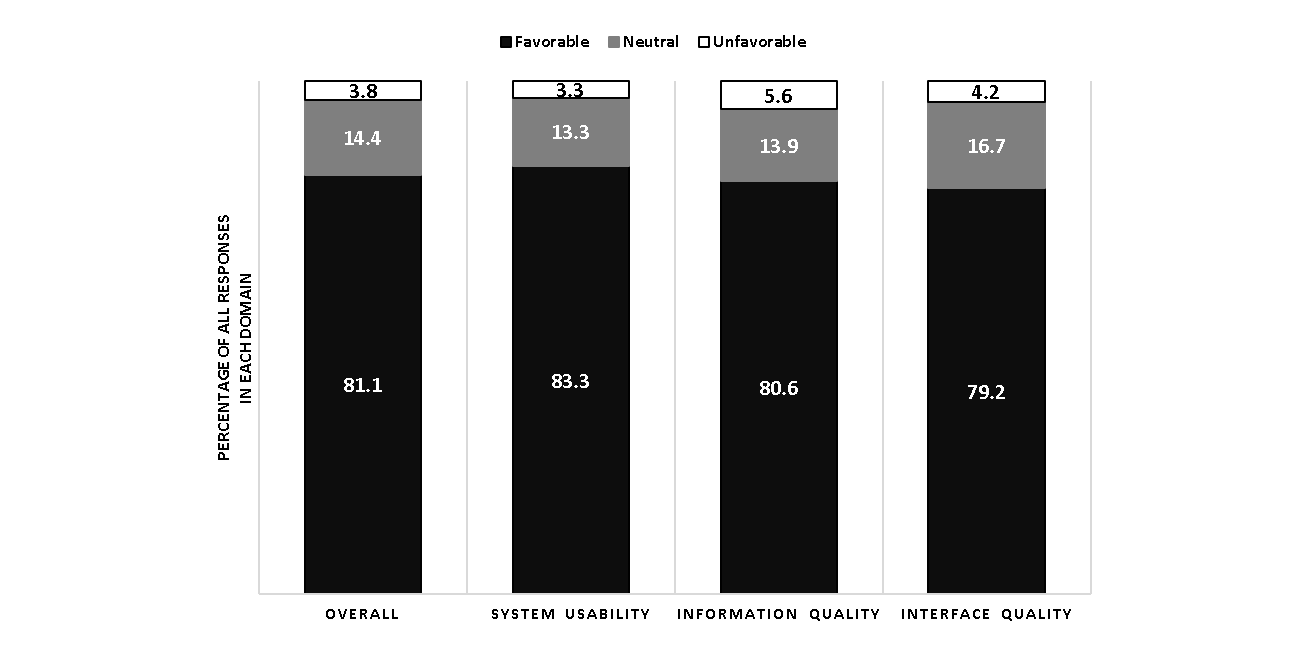
Percent of responses categorized as favorable, unfavorable, or neutral within each domain from the postimplementation computer usability scale questionnaire (respondents=12).

Fifty computer systems usability questionnaires were distributed to clinicians who had the opportunity to use the tool in clinical practice during the 2-month period. We received 24% (12/50) responses. The questionnaire was completed by 11% (1/9) residents, 17% (4/24) fellows, and 42% (7/17) attending physicians. A summary of user usability feedback is shown in Figure 2.

## Discussion

### Principal Findings

The first part of our study estimates the time-drain of manual SOFA score calculation with a modern EMR system and describes an attempt to mitigate these inefficiencies with an EMR-integrated automated SOFA score calculator created through a user-centered design process. Our time-motion analysis found that the current time required for manual calculation using a modern EMR has improved compared with a study performed 5 years prior [23]. However, these efficiencies may be obscured by the need for repeated calculation under the new sepsis definition. Real-world usage would likely dictate more frequent recalculation and consequently automation would be more desirable as the cumulative time requirements increase.

The second part of our study describes the iterative, user-centered design process for an EMR-integrated automated calculator “app” for the SOFA score. Clinician stakeholders worked closely with developers throughout the rapid UX development process. The resulting interface was favorable to clinician end users in all three usability domains assessed (system usability, information quality, and information quality).

### Comparison With Prior Work

Several other clinical scores have been automated for clinical practice—examples include APACHE II [32,33], APACHE IV [34], CHA_2_ DS_2_-VASc [35], Charlson comorbidity score [36], and early warning systems [37]. These studies primarily focused on algorithm validation rather than information delivery. The information delivery needs for clinicians using these clinical scores depends on the clinical context; many clinical scoring systems are used at decision points in patient care and clinical practice guidelines (CPG). Clinicians’ poor CPG adherence has been recognized for many years [38]. Consequently, user-centered design processes have been utilized to improve CPG adherence though clinical decision support—ranging from surgical pathways to guideline implementation—with favorable results [26,39-42].

### Future Directions

Future demand for SOFA score calculation in clinical practice may be dependent on policy from the Centers for Medicare and Medicaid Services (CMS), which is still recommending the use of the previous definition of sepsis outlined in the Severe Sepsis or Septic Shock Early Management (SEP-1) bundle because of concerns about increasing cases of missed sepsis under the new definition [16,17,19]. CMS adoption of the new sepsis definition would likely spur a significant increase in the usage of the SOFA score by linking quality metrics and payments. Because of the time-cost of score calculation in an otherwise busy clinical setting, manual SOFA score recalculation may only be performed after the clinician has already suspected new onset sepsis due to physiologic changes noted at the bedside. In this situation, application of the ΔSOFA definition (≥2 over baseline) would be confirmatory and not predictive—counter to the Surviving Sepsis Campaign’s goal to improve early recognition of sepsis [43]. However, by automating the SOFA score calculation process and repeating the calculation as new clinical information becomes available, the ΔSOFA criteria could effectively function as a sepsis sniffer. Further studies would be needed to compare the effectiveness of the ΔSOFA criteria as a “sepsis sniffer” against other “black box” sepsis detection algorithms being developed [37,44-48]. The application of the ΔSOFA criteria as a “sepsis sniffer” does have promise—a recent retrospective study demonstrated that SOFA has greater discrimination for in-hospital mortality in critically-ill patients than either quick SOFA (qSOFA) or systemic inflammatory response syndrome (SIRS) criteria [49].

The pairing of the automated SOFA calculator algorithm with the user-centered UX design may hold advantages over these machine-learning based “black box” algorithms—our underlying algorithm is based on a familiar, well-validated clinical score and the visualization of each SOFA component allows clinicians to “look under the hood” to explore the source data behind each item’s value. The ability to verify the source data within the UX reflected the information needs of our clinician stakeholders identified during the agile software development process. With the “black box” algorithms of artificial neural networks and other machine learning techniques, a comparable level of transparency is not possible. Finally, traditional externally validated clinical scores, like SOFA, may be more generalizable than machine-learning algorithms [50]. The external validity of these machine learning algorithms is dependent on the diversity of the data sources used for training and cross-validation, whereas traditional clinical scores adopted into CPGs have already been externally validated. Consequently, researchers may have an opportunity to translate and distribute traditional clinical scoring models as automated computerized algorithms through interoperability platforms.

The emerging “substitutable medical apps, reusable technology” (SMART) on “fast healthcare interoperability resources” (FHIR) interoperable application platform is a promising avenue to bridge the gap between standalone applications and EMR integration [51]. Additionally, the platform offers a means to reduce the 17-year gap between clinical-knowledge generation and widespread usage [52]. Under this platform, interoperable applications can be developed and widely distributed like popular mobile phone apps. Calculator apps and other forms of clinical decision support are currently being “beta-tested” on this platform [53]. In the future, researchers developing clinical scores or computer-assisted decision algorithms may be encouraged to develop similar interoperable applications. In the “app” domain, whether on a mobile phone or integrated into the EMR, usability is an important feature that must be balanced with functionality to encourage widespread adoption. The agile development process described in this paper involved clinicians in the development process early and often, leading to an EMR-integrated “app” that met both clinician information and usability needs within a concise 2-week timeline.

### Limitations

The primary limitation of this study is that the clinician stakeholders are from a single institution and their needs might not match the needs of clinicians elsewhere. However, a similar user-centered design and evaluation process could be utilized at other institutions to create and customize a similar tool. Second, clinician survey response rate was low. We aimed to include residents, fellows, and critical care attending physicians with exposure to the tool to obtain perspectives from a wide variety of clinical roles. However, nearly all survey responses were provided by critical care attending physicians and fellows. The tool appeared to meet the usability needs of these content experts.

### Conclusions

The incorporation of SOFA scoring into the sepsis definition potentially adds about 1 min per patient (calculation) to an intensive care clinicians’ workload—an amount that is compounded when recalculation is performed multiple times daily to confirm if ΔSOFA criteria have been met. This added workload can be eliminated through automated information retrieval and display. To generate the information display for an EMR-integrated automated SOFA score calculator, we utilized a user-centered agile design process that resulted in a user interface with >75% of usability features receiving favorable ratings across the system usability, information quality, and interface quality usability domains. Usability evaluations are important as clinical decision support algorithms are translated into EMR-integrated applications.

## Abbreviations

CMS: Centers for Medicare and Medicaid Services
CPG: clinical practice guideline
EMR: electronic medical record
FHIR: fast healthcare interoperability resources
GUI: graphical user interface
ICU: intensive care unit
qSOFA: quick SOFA
SEP-1: Severe Sepsis or Septic Shock Early Management Bundle
SIRS: systemic inflammatory response syndrome
SMART: substitutable medical apps, reusable technology
SOFA: Sequential Organ Failure Assessment
UX: User Interface
